# Effect of noise isolation during general anaesthesia on the incidence of moderate-to-severe pain after major abdominal surgery: multicentre randomized clinical study

**DOI:** 10.1093/bjsopen/zrag035

**Published:** 2026-05-28

**Authors:** Fang Xu, Dong Chen, Cong Wang, Yi Yang, Yingcai Wu, Shuai You, Guangyou Duan

**Affiliations:** Department of Anesthesiology, The Second Affiliated Hospital, Chongqing Medical University, Chongqing, China; Department of Anesthesiology, Chonggang General Hospital, Chongqing, China; Department of Anesthesiology, Perioperative and Pain Medicine, The First Affiliated Hospital of Zhengzhou University, Zhengzhou, China; Department of Anesthesiology, The Second Affiliated Hospital, Chongqing Medical University, Chongqing, China; Department of Anesthesiology, The Second Affiliated Hospital, Chongqing Medical University, Chongqing, China; Department of Anesthesiology, Shapingba Hospital Affiliated to Chongqing University (Shapingba District People's Hospital of Chongqing), Chongqing, China; Department of Anesthesiology, The Second Affiliated Hospital, Chongqing Medical University, Chongqing, China

**Keywords:** postoperative pain management, major surgery, intraoperative environment, noise reduction

## Abstract

**Background:**

Postoperative pain remains a major challenge following major abdominal surgery. Noise in the operating room is a modifiable stressor, and the efficacy of targeted noise isolation requires prospective investigation. This study investigated the effects of intraoperative noise isolation on the incidence of moderate-to-severe postoperative pain.

**Methods:**

This multicentre randomized clinical trial assessed patients who underwent elective major abdominal surgery under general anaesthesia in four medical centres in China between April, 2024 and May, 2025. Following anaesthesia induction, patients were randomized to either wear noise-cancelling headphones or not (control). The primary outcome of this study was the incidence of moderate-to-severe pain (numeric rating scale (NRS) score ≥ 4) within the 24-hour (h) period after surgery. Secondary outcomes included the incidence of moderate-to-severe pain, cumulative postoperative pain NRS scores within 48 h after surgery, and the consumption of analgesic drugs within 24 and 48 h after surgery.

**Results:**

In all, 304 patients were enrolled and randomized; 302 patients were included in the final analysis (150 in the noise isolation group, 152 in the control group). The incidence of moderate-to-severe pain was higher in the control than noise isolation group within 24 h after surgery (49 *versus* 23%, respectively; relative risk (RR) 0.47; 95% confidence interval (c.i.) 0.33 to 0.65; *P*  *<* 0.001) and within 48 h after surgery (51 *versus* 25%, respectively; RR 0.48; 95% c.i. 0.35 to 0.66; *P* < 0.001). During the 24-h and 48-h periods after surgery, the number of patient-controlled intravenous analgesia boluses was significantly higher in the control group, which also had a higher extra analgesia requirement and increased total analgesic consumption compared with the noise isolation group. The cumulative and maximum rest and movement pain scores were higher in the control than noise isolation group during the 48-h period after surgery.

**Conclusions:**

Intraoperative noise isolation was found to be an effective, safe, and non-invasive preventive intervention that significantly lowered the incidence of moderate-to-severe pain after major abdominal surgery, arguing for its integration into standard multimodal analgesic strategies. Registration number: NCT06316440 (http://www.clinicaltrials.gov).

## Teaser

This multicentre randomized clinical study in 304 adults investigated the effect of noise isolation during surgery on the incidence of moderate-to-severe pain. In the 24-hour period after surgery, the incidence of moderate-to-severe pain was significantly lower in the noise isolation than control group (23 *versus* 49%). Intraoperative noise isolation was found to be an effective, safe, and non-invasive preventive intervention that significantly lowered the incidence of moderate-to-severe pain after major abdominal surgery.

## Introduction

Globally, over 300 million surgical procedures are performed annually^1^, with approximately 70 million in-patients undergoing surgery each year in China^2^. Within this vast clinical landscape, postoperative pain stands as the most prevalent complication. Inadequate pain control after surgery can severely hinder recovery by delaying early mobilization, increasing complication rates, extending the length of hospital stay, and imposing a substantial economic burden on healthcare systems^3,4^. Therefore, enhancing postoperative pain management is vital. Although a range of perioperative measures are used to mitigate pain, their efficacy is often limited, with the incidence of moderate-to-severe pain remaining as high as 40–60% globally^2^, and 30–55% after abdominal surgery^5^ . This persistent burden underscores the critical need for more effective analgesic strategies.

Currently, pharmacological treatments, including opioid analgesics, non-steroidal anti-inflammatory drugs, and local anaesthetics, are the mainstay of postoperative pain therapy^6^. However, these pharmacological treatments can lead to a range of side effects, such as nausea, vomiting, itching, urinary retention, hepatorenal toxicity, and gastrointestinal ulcers^7–9^. In recent years, studies have evaluated non-pharmacological and non-invasive methods to treat postoperative pain, including music therapy^10^, acupuncture^11^ and psychological interventions^12^. However, most of these methods are used after pain has already occurred. Currently, studies focusing on targeted preventive interventions for the causes of postoperative pain are lacking.

Previous work identified high-level intraoperative noise as a significant contributor to increased postoperative pain and analgesic requirements in patients undergoing abdominal surgery under general anaesthesia^13^. Building upon this finding, a subsequent exploratory randomized clinical trial (RCT) demonstrated that intraoperative noise isolation via noise-cancelling headphones provided a simple, non-invasive intervention capable of alleviating postoperative pain and reducing total opioid consumption in this patient population^14^. However, the generalizability of these findings was constrained by the limited sample size, single-centre study design, and its reliance on intravenous-inhalational anaesthesia, a technique that contrasts with the total intravenous anaesthesia approach more common in international practice. To address the limitations of these previous studies and definitively establish the efficacy of this intervention, a large-scale multicentre trial is warranted. Thus, the aim of the present study was to clarify the impact of noise isolation during major abdominal surgery on the incidence of moderate-to-severe postoperative pain.

## Methods

### Research design

This was a prospective multicentre RCT involving four tertiary hospitals in China: The Second Affiliated Hospital of Chongqing Medical University; The Shapingba Hospital, Chongqing University; The First Affiliated Hospital of Zhengzhou University; and Chonggang General Hospital. The study protocol was formulated in accordance with the CONSORT guidelines for RCTs^15^, with participants enrolled sequentially at a single centre. The study was approved by the Ethics Committee of The Second Affiliated Hospital of Chongqing Medical University (Approval no. 140 2023 (KELUN-SHEN)) and was registered at ClinicalTrials.gov (ID NCT06316440). The study was conducted between April 2024 and May 2025. All patients provided informed consent before inclusion. The protocol for this research available in *Appendix S1*.

### Inclusion and exclusion criteria

The inclusion criteria were as follows: age between 18 and 70 years, with no sex restrictions; American Society of Anesthesiologists (ASA) Grades I–III; patients undergoing elective major abdominal surgery under general anaesthesia (with an operation time of ≥ 2 hours (h)); and voluntary acceptance of patient-controlled intravenous analgesia (PCIA) and signing of the informed consent form. Patients with a history of severe disease and ASA Grade ≥ IV, those with hearing problems, those who required mechanical ventilation or received epidural catheters or other types of regional anaesthesia after the operation, those with chronic pain before surgery and/or those who had been taking painkillers for a long time, and those who were unable to cooperate with the research for any reason were excluded from the study. Patients were also excluded if they voluntarily terminated trial participation during the study and had missing data that affected the assessment of the validity of the intervention.

### Randomization and blinding

In this study, a simple central randomization method was used. Computer-generated numbers from 1 to 304 were randomly placed in 304 opaque, numbered, and sealed envelopes. Odd and even computer-generated numbers were designated as the experimental and control groups, respectively. All sites participating in the study reported eligible patients to the principal investigator (PI) at the Department of Anesthesiology of The Second Affiliated Hospital of Chongqing Medical University before their surgery. The study PI assigned patients to either the control or intervention group based on the random numbers in the envelopes. Noise-blocking measures for patients were initiated after the induction of general anaesthesia and stopped at the end of surgery. In this study, none of the patients was aware of their group allocation. Data (baseline and postoperative) were collected by trained professionals who were not involved in the anaesthesia process and were blinded to group assignment. Anaesthesiologists and surgeons involved in the anaesthesia and surgery did not participate in the collection, entry, or analysis of the research data.

### Anaesthesia, analgesia, and intervention

On the day before surgery, enrolled patients underwent routine preoperative visits. In addition to signing the anaesthesia consent form, patients were informed about the study procedures and precautions and signed the informed consent form for the study. The Huaxi Emotional-distress Index (HEI)^16^ was used to assess patients’ emotional status. The HEI is used to screen for adverse emotions (anxiety and depression) in hospitalized patients. It consists of nine items, scored using a five-point Likert scale, as follows: 0, none at all; 1, occasionally; 2, some of the time; 3, most of the time; and 4, all of the time. Higher scores indicate more severe adverse emotions. Patients were also instructed on how to use the numeric rating scale (NRS) for pain assessment and educated on the use of the PCIA pump.

On the day of surgery, after the patient had entered the operating room, routine electrocardiogram, pulse oximetry, heart rate, and invasive blood pressure monitoring were initiated and maintained. The depth of anaesthesia was monitored continuously using a Bispectral Index (BIS™) monitor (Medtronic, Dublin, Ireland)until the end of the surgery. The Sima Instrument AR844 noise meter (Dongguan Wanchuang Electronic Products, Dongguan, China) was used to monitor noise levels. The noise meter recorded data at a frequency of once per second and had a measurable range of 0–130 dB and continuously monitored noise levels from the start of anaesthesia (induction) to the end of anaesthesia (cessation of anaesthetic infusion), which was defined as the noise during general anaesthesia in the present study. The noise meter measured A-weighted sound (ambient noise) levels during the entire period of general anaesthesia^17^. After data collection, the data were exported from the device-associated software (VoicelAB Real Time Measure, provided by the manufacturer) to calculate the mean A-weighted noise intensity during the entire period of general anaesthesia.

General anaesthesia was administered by an experienced anaesthesiologist. Patients in the control and noise isolation groups underwent an identical anaesthesia protocol. All patients included in the study underwent rapid-sequence induction of anaesthesia with midazolam (0.04 mg/kg), sufentanil (0.3–0.5 µg/kg), propofol (2–2.5 mg/kg), and rocuronium 0.6 mg/kg. Anaesthesia was maintained with total intravenous anaesthesia using a combination of remifentanil (0.1–0.2 µg per kg per min) and propofol (4–12 mg per kg per h) infusion, with the depth of anaesthesia maintained at a Bispectral Index (BIS) value between 40 and 60. Heart rate and blood pressure were controlled within 20% of baseline values. Sufentanil and rocuronium were administered in bolus doses as required during surgery. At the end of the surgery, ondansetron 8 mg was administered.

In the intervention group, noise-cancelling headphones (3M™ PELTOR™ X5A; Wroclaw, Poland) were worn continuously from the end of anaesthetic induction until the completion of surgery. According to the ISO 4869, these headphones have a single-number noise reduction rating of 37 dB. To ensure proper fit, the headband and ear cups were adjusted to each patient's head and facial morphology, with the ear cushions positioned to cover the ears and the headband resting over the top of the head. Throughout the trial, patient positioning and headphone placement were closely monitored to prevent displacement and avoid compression of the auricle or scalp. In the control group, no noise-cancelling headphones were used after anaesthesia induction and intubation. After surgery, all patients received bilateral transversus abdominis plane blocks guided by ultrasound using 40 ml of 0.375% ropivacaine hydrochloride for postoperative analgesia, and a PCIA pump was connected. The PCIA pump contained hydromorphone 0.15 mg/kg plus 0.9% saline, with a total volume of 150 ml. The pump settings were as follows: loading dose 2 ml; background infusion rate 2 ml/h; patient-controlled dose 2 ml; and lock-out time 15 min. Additional analgesia was provided by the surgical team with non-steroidal anti-inflammatory drugs, such as flurbiprofen axetil, according to patients’ clinical needs.

### Study outcomes

The primary outcome of this study was the incidence of moderate-to-severe postoperative pain (NRS ≥ 4) assessed separately at rest and upon movement within the first 24 h after surgery. Secondary outcomes were the incidence of moderate-to-severe pain within 48 h after surgery, the cumulative postoperative pain NRS scores within 48 h after surgery, and the consumption of analgesic drugs within 24 and 48 h after surgery.

The primary outcome measure was the maximum pain NRS score experienced by the patient within the 48-h period after surgery (ranging from 0 to 10, where 0 indicates no pain at all and 10 indicates the most severe pain; the higher the score, the more severe the pain). Patients were assessed at 6, 12, 24, and 48 h after surgery to record the maximum resting and movement-evoked pain experienced 0–6, 6–12, 12–24, and 24–48 h after surgery. In this study, resting pain refers to pain experienced while lying still in bed, whereas movement-evoked pain refers to pain experienced during activities such as turning over or coughing. Other recorded parameters included general demographic information before surgery, the patient's emotional state, ASA grade, the type of surgery, the duration of surgery, intraoperative medication use, and postoperative nausea, vomiting, urinary retention, and dizziness.

### Statistical analysis

In this study, the primary outcome was the incidence of moderate-to-severe pain within the 24-h period after surgery. Based on previous clinical observations, the incidence of moderate-to-severe pain within 24 h after abdominal surgery was approximately 50%. The hypothesis in this study was that the incidence of moderate-to-severe pain within 24 h after abdominal surgery would be reduced to 30% after the implementation of noise isolation measures. Using the sample size calculation software PASS-2021 (NCSS, LLC, Kaysville, US), according to a 1 : 1 parallel controlled difference test study design, with a significance level of 0.05 and power of 0.9, the minimum required sample size was 121 patients per group. Considering a 20% dropout rate, it was determined that 152 patients needed to be included in each group. Thus, the aim was to include 304 patients, in total, in the present study.

Statistical analyses were performed using SPSS^®^ 26.0 (IBM, Armonk, NY, USA), with *P* < 0.05 (two-tailed) was considered to be statistically significant. The primary outcome was compared according to the modified intention to-treat principle and included patients who had provided consent, undergone randomization, and completed the 48-h postoperative follow-up. The incidence of moderate-to-severe pain within 24 h after surgery was compared using χ^2^ tests, with differences between groups expressed as a relative risk (RR) with 95% confidence interval (c.i.). A post hoc sensitivity analysis was also performed by imputing missing primary endpoint data. The worst outcome to participants in the noise isolation group was assigned to missing data.

Secondary outcomes were analysed as follows. Continuous variables were compared using an independent *t* test or Mann–Whitney test, whereas categorical variables were analysed using χ^2^ tests, continuity-corrected χ^2^ tests, or Fisher’s exact test. Mean differences (MDs) or RRs with 95% c.i. were also calculated. Univariate logistic regression analyses were performed to determine the effects of sex (female *versus* male), age group (≥ 60 *versus* < 60 years), body mass index group (BMI; ≥ 28 *versus* < 28 kg/m^2^), ASA grade (III *versus* II), surgery type (gynaecological *versus* gastrointestinal), HEI (≥ 9 *versus* < 9), surgical method (open *versus* endoscopic), cancer surgery (yes *versus* no), and study group (noise isolation *versus* control) on the incidence of moderate-to-severe pain within the 24-h period after surgery. Multiple logistic regression analysis was then performed using the factors with *P* < 0.1 in the univariate analysis. Subgroup analyses were also performed to compare differences between the noise isolation and control groups.

## Results

**Fig. 1:**
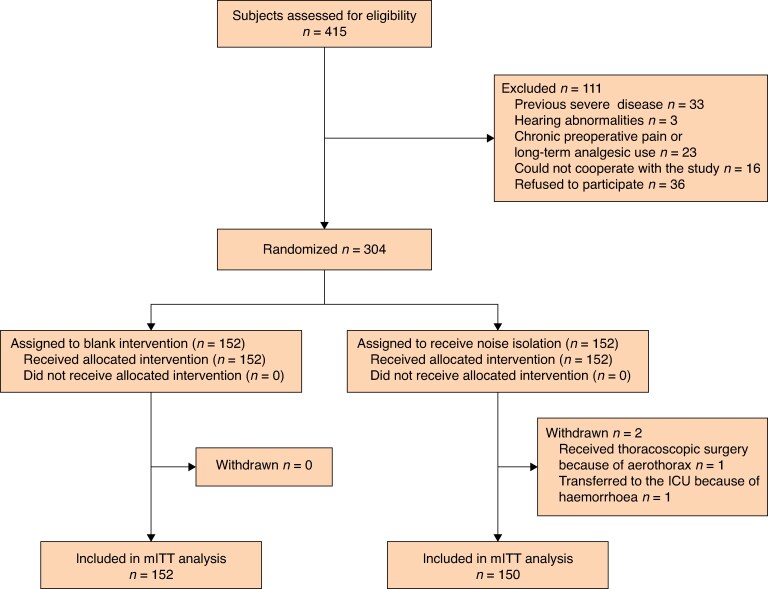
Flow chart of study patients ICU, intensive care unit; mITT, modified intention-to-treat.

As shown in *Fig. 1*, 302 patients were included in the final analysis (150 in the noise isolation group and 152 in the control group). Baseline and intraoperative characteristics of patients in the noise isolation and control groups are presented in *Table 1*. Two variables differed significantly between the two groups: the mean noise intensity was higher in the control group than in the noise isolation group (mean(standard deviation (s.d.)) 64.5(2.8) *versus* 63.9(2.5) dB, respectively; MD −0.62 dB; 95% c.i., −1.23 to −0.16 dB; *P*  *=* 0.044), and the proportion of patients undergoing laparoscopic surgery was lower in the control than noise isolation group (82 *versus* 90%, respectively; RR 2.03; 95% c.i. 1.04 to 3.98; *P* = 0.036). All other variables were comparable between the two groups (*Table 1*).

**Table 1:** Baseline and intraoperative data for patients in the noise isolation and control groups

	Control group (*n* = 152)	Noise isolation group (*n* = 150)
Age (years), mean(s.d.)	53.2(9.5)	53.0(9.2)
**Age group**		
≥ 60 years	40 (26%)	40 (27%)
< 60 years	112 (74%)	110 (73%)
**Sex**		
Male	40 (26%)	52 (35%)
Female	112 (74%)	98 (65%)
Height (cm), mean(s.d.)	160.1(7.8)	160.7(6.9)
Weight (kg), mean(s.d.)	62.2(11.5)	63.6(10.6)
BMI (kg/m^2^), mean(s.d.)	24.2(3.6)	24.6(3.6)
**BMI group**		
BMI ≥ 28 kg/m^2^	21 (14%)	26 (17%)
BMI < 28 kg/m^2^	131 (86%)	124 (83%)
ASA grade		
Grade II	141 (93%)	132 (88.0%)
Grade III	11 (7%)	18 (12%)
HEI	2.0 (1.0–4.0)	2.0 (1.0–4.0)
**HEI group**		
HEI ≥ 9	6 (4%)	2 (1%)
HEI < 9	146 (96%)	148 (99%)
Surgery duration (h), mean(s.d.)	3.3(1.3)	3.2(1.1)
Blood loss (ml), median (i.q.r.)	50 (50–150)	50 (50–100)
**Total intravenous anaesthesia**		
Remifentanil (mg), median (i.q.r.)	1.9 (1.4–2.6)	2.0 (1.4–2.6)
Sufentanil (µg), mean(s.d.)	38.2(8.7)	40.3(9.1)
Mean noise intensity (dB), mean(s.d.)	64.5(2.8)	63.9(2.5)
**Surgery type**		
Gynaecological	85 (56%)	79 (53%)
Gastrointestinal	67 (44%)	71 (47%)
**Surgical method**		
Laparoscopic	124 (82%)	135 (90%)
Open	28 (18%)	15 (10%)
**Cancer surgery**		
Yes	75 (49%)	81 (54%)
No	77 (51%)	69 (46%)
**Study site**		
Site 1	49 (32%)	51 (34%)
Site 2	17 (11%)	24 (16%)
Site 3	31 (20%)	26 (17%)
Site 4	55 (36%)	49 (33%)

Values are *n* (%) unless otherwise indicated. s.d., standard deviation; BMI, body mass index; ASA, American Society of Anesthesiologists; HEI, Huaxi Emotional-distress Index; h, hours.

The study outcomes in the noise isolation and control groups are presented in *Table 2*. The primary outcome of the study, namely the incidence of moderate-to-severe pain, was higher in the control group than in the noise isolation group during the 24-h period after surgery (49 *versus* 23%, respectively; RR 0.47; 95% c.i. 0.33 to 0.65; *P* < 0.001). A significant between-group difference was observed in secondary outcomes during the first 24 h after surgery. Although the number of PCIA boluses administered within 24 h after surgery was identical in both groups (median 0.0 (interquartile range (i.q.r.) 0.0–2.0), the Mann–Whitney *U* test, which compares the overall rank distribution, indicated a statistically significant difference between the two groups (MD −0.60; 95% c.i. −1.20 to −0.01; *P* = 0.046). Significantly more patients in the control group than noise isolation group in the required additional analgesia (20 *versus* 8%, respectively; RR 0.39; 95% c.i. 0.21 to 0.73; *P* = 0.003) and the mean(s.d.) total analgesic consumption was higher in the control group (36(10.8) *versus* 33.8(8.0) mg morphine equivalent; MD −2.23 mg morphine equivalent; 95% c.i. −4.38 to −0.09 mg morphine equivalent; *P* = 0.041). During the 48-h period after surgery, compared with the noise isolation group, the control group had a higher incidence of moderate-to-severe pain (51 *versus* 25%; RR 0.48; 95% c.i. 0.35 to 0.66; *P*  *<* 0.001), a higher number of button presses of the PCIA pump (median 1.0 (i.q.r. 0.0–3.0) *versus* 0.0 (i.q.r. 0.0–2.0); MD 0.0; 95% c.i. 0.00 to 0.00; *P* = 0.026), and a higher proportion of patients requiring additional analgesia (22% *versus* 9%; RR 0.43; 95% c.i. 0.24 to 0.77; *P* = 0.004). There was no significant difference in total postoperative analgesic consumption between the control and noise isolation groups (mean(s.d.) 65(19.8) *versus* 61.5(16.3) mg morphine equivalent, respectively; MD: −3.53 mg morphine equivalent; 95% c.i. −7.64 to 0.58 mg morphine equivalent; *P* = 0.093; *Table 2*).

**Table 2:** Study outcomes in the noise isolation and control groups

	Control group (*n* = 152)	Noise isolation group (*n* = 150)	Estimated effect*	*P*
**Moderate-to-severe pain**				
During the 24 h after surgery	74 (49%)	34 (23%)	RR: 0.47 (0.33, 0.65)	< 0.001
During the 48 h after surgery	78 (51%)	37 (25%)	RR: 0.48 (0.35, 0.66)	< 0.001
**No. PCIA boluses**				
During the 24 h after surgery, median (i.q.r.)	0 (0–2)	0 (0–2)	MD: −0.60 (−1.20, −0.01)	0.046
During the 48 h after surgery, median (i.q.r.)	1 (0–3)	0 (0–2)	MD: −0.81 (−1.55, −0.07)	0.026
**Extra analgesia required**				
During the 24 h after surgery	31 (20%)	12 (8%)	RR: 0.39 (0.21, 0.73)	0.003
During the 48 h after surgery	33 (22%)	14 (9%)	RR: 0.43 (0.24, 0.77)	0.004
**Total postoperative analgesia consumption† (mg)**				
At 24 h, mean (s.d.)	36 (10.8)	33.8 (8.0)	MD: −2.23 (−4.38, −0.09)	0.041
At 48 h, mean (s.d.)	65 (19.8)	61.5 (16.3)	MD: −3.53 (−7.64, 0.58)	0.093
Postoperative nausea and vomiting	28 (19%)	20 (13%)	RR: 0.71 (0.42, 1.19)	0.190
LOS (days), median (i.q.r.)	13.5 (9.0–18.0)	13.0 (9.0–16.0)	MD: −1.0 (−2.0, 0.0)	0.353

Values are *n* (%) unless otherwise indicated. *Values in parentheses are 95% confidence intervals. †Calculated as the equivalent dose of morphine. RR, relative risk; h, hours; PCIA, patient-controlled intravenous analgesia; s.d., standard deviation; i.q.r., interquartile range; MD, mean difference; LOS, length of hospital stay.

**Fig. 2:**
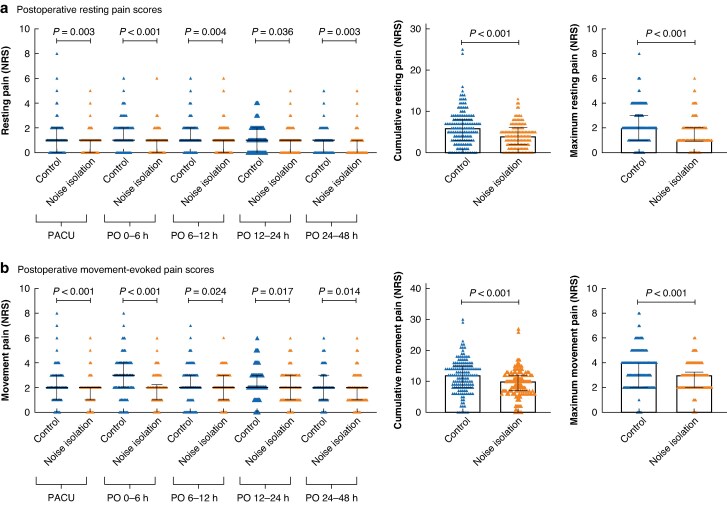
Comparisons of postoperative pain scores at specific timepoints between the noise isolation and control groups **a** Postoperative resting pain scores at different timepoints and cumulative and maximum postoperative resting pain scores during the 48-h period after surgery. **b** Postoperative movement-evoked pain scores at different timepoints and cumulative and maximum postoperative movement-evoked pain scores during the 48-h period after surgery. Data were showed as median with i.q.r. NRS, numeric rating scale; PACU, post-anesthesia care unit; PO, postoperative timepoint; i.q.r., interquartile range; h, hours.

Comparisons of postoperative pain scores at specific timepoints between the noise isolation and control groups are shown in *Fig. 2*. Based on the NRS, the postoperative resting pain scores were significantly higher in the control group than in the noise isolation group at all timepoints, as were the movement-evoked pain scores. In the 48-h period after surgery, the control group had higher cumulative resting pain scores (median 6.0 (i.q.r. 3.0–8.0) *versus* 4.0 (i.q.r. 2.0–6.0); MD –2.0; 95% c.i. −3.00 to −1.00; *P*  *<* 0.001) and maximum resting pain scores (median 2.0 (i.q.r. 1.0–3.0) *versus* 1.0 (i.q.r. 1.0–2.0); MD –1.0; 95% c.i. −1.00 to 0.00; *P*  *<* 0.001) than noise isolation group. The control group also had higher cumulative movement pain scores (median 12.0 (i.q.r. 8.0–15.0) *versus* 10.0 (i.q.r. 7.0–12.0); MD –2.0; 95% c.i. −3.00 to −1.00; *P*  *<* 0.001) and maximum movement pain scores (median 4.0 (i.q.r. 2.0–4.0) *versus* 3.0 (i.q.r. 2.0–3.25); MD –1.0; 95% c.i. −1.00 to 0.00; *P*  *<* 0.001) than the noise isolation group in the 48-h period after surgery.

Multivariate regression analysis (*Table 3*) revealed that the independent factors affecting the incidence of moderate-to-severe pain during the 24-h period after surgery were the surgical method (RR 2.35 (95% c.i. 1.23 to 4.52) for open *versus* laparoscopic surgery; *P*  *=* 0.010) and group assignment (RR 0.31 (95% c.i. 0.19 to 0.51) for noise isolation *versus* control; *P* < 0.001). Furthermore, after adjustment, both surgery type and surgical method were significant affecting the incidence of moderate-to-severe pain in the 24 h after surgery (RR 0.49 (95% c.i. 0.29 to 0.83), for gynaecological *versus* gastrointestinal surgery (*P* = 0.007); RR 2.68 (95% c.i. 1.31 to 5.47) for open *versus* laparoscopic surgery (*P* = 0.007)). Crucially, in this adjusted model, the association for group assignment remained highly significant (RR 0.31 (95% c.i. 0.19 to 0.52) for noise isolation *versus* control; *P* < 0.001).

**Table 3:** Effects of baseline factors on the risk of moderate-to-severe pain during the 24 h after surgery

	RR*	*P*	Adjusted RR*	*P*
Sex (female *versus* male)	1.32 (0.79, 2.18)	0.286		
Age group (≥ 60 *versus* < 60 years)	1.11 (0.65, 1.88)	0.705		
BMI group (≥ 28 *versus* < 28 kg/m^2^)	1.09 (0.57, 2.11)	0.789		
ASA grade (III *versus* II)	1.10 (0.50, 2.44)	0.798		
Surgery type (gynaecological *versus* gastrointestinal)	0.64 (0.40, 1.03)	0.066	0.49 (0.29, 0.83)	0.007
HEI (≥ 9 *versus* < 9)	0.93 (0.22, 3.95)	0.917		
Surgical method (open *versus* laparoscopic)	2.35 (1.23, 4.52)	0.010	2.68 (1.31, 5.47)	0.007
Cancer surgery (yes *versus* no)	1.43 (0.89, 2.30)	0.697		
Group (noise isolation *versus* control)	0.31 (0.19, 0.51)	<0.001	0.31 (0.19, 0.52)	<0.001

^∗^Values in parentheses are 95% confidence intervals. RR, relative risk; BMI, body mass index; ASA, American Society of Anesthesiologists; HEI, Huaxi Emotional-distress Index.

Subgroup analysis revealed that the incidence of moderate-to-severe pain was consistently lower in the noise isolation than control group across most subgroups (*Table 4*). However, this difference was not observed in the subgroups of patients aged ≥ 60 years or those who underwent open surgery, where no significant intergroup differences were found (*Table 4*).

**Table 4:** Subgroup analysis for the incidence of moderate-to-severe pain according to noise isolation during general anaesthesia

	Moderate-to-severe pain	Relative risk*	*P*
**Sex**			
Male		0.52 (0.31, 0.87)	0.011
Control (*n* = 40)	22 (55%)		
Noise isolation (*n* = 52)	15 (29%)		
Female		0.42 (0.27, 0.65)	< 0.001
Control (*n* = 112)	38 (45%)		
Noise isolation (*n* = 98)	13 (17%)		
**Age group**			
< 60 years		0.40 (0.26, 0.61)	< 0.001
Control (*n* = 112)	56 (50%)		
Noise isolation (*n* = 110)	22 (20%)		
≥ 60 years		0.67 (0.37, 1.19)	0.166
Control (*n* = 40)	18 (45%)		
Noise isolation (*n* = 40)	12 (30%)		
**BMI group**			
< 28 kg/m^2^		0.51(0.36, 0.73)	< 0.001
Control (*n* = 131)	62 (47%)		
Noise isolation (*n* = 124)	30 (24%)		
≥ 28 kg/m^2^		0.27(0.10, 0.71)	0.003
Control (*n* = 21)	12 (57%)		
Noise isolation (*n* = 25)	4 (15%)		
**Surgery type**			
Gynaecological		0.37 (0.21, 0.64)	< 0.001
Control (*n* = 85)	38 (45%)		
Noise isolation (*n* = 79)	13 (17%)		
Gastrointestinal		0.55 (0.36, 0.84)	0.004
Control (*n* = 67)	36 (54%)		
Noise isolation (*n* = 71)	21 (30%)		
**Surgical method**			
Open		0.82(0.44, 1.53)	0.512
Control (*n* = 28)	16 (57%)		
Noise isolation (*n* = 15)	7 (47%)		
Laparoscopic		0.43(0.29, 0.63)	< 0.001
Control (*n* = 124)	58 (47%)		
Noise isolation (*n* = 135)	27 (20%)		
**Cancer surgery**			
Yes		0.67 (0.45, 0.99)	0.043
Control (*n* = 75)	36 (48%)		
Noise isolation (*n* = 81)	26 (32%)		
No		0.23 (0.12, 0.47)	< 0.001
Control (*n* = 77)	38 (49%)		
Noise isolation (*n* = 69)	8 (12%)		

Values are *n* (%) unless otherwise indicated. *Values in parentheses are 95% confidence intervals. BMI, body mass index.

## Discussion

This study revealed that, compared with the control group, patients with intraoperative noise isolation had a significantly reduced incidence of moderate-to-severe pain within 24 h after major abdominal surgery. Beyond this positive outcome for the primary endpoint, significant reduction were seen in the noise isolation group in the secondary endpoints of incidence of moderate-to-severe pain within 48 h after surgery, cumulative pain scores, postoperative analgesic consumption, and additional analgesic requirements within 24 and 48 h after surgery.

Compared with the earlier single-centre exploratory study^14^, the present study has more participating centres and a larger sample size. Moreover, this study used the incidence of moderate-to-severe pain rather than single-timepoint pain scores as the primary endpoint, an outcome measure that holds greater clinical significance. In this study, the incidence of moderate-to-severe pain within 24 h after major abdominal surgery in the control group was 49%, consistent with previously reported findings^2^. For the first time, the present multicentre RCT has demonstrated that isolating environmental noise during general anaesthesia reduces the risk of early postoperative moderate-to-severe pain (observed incidence: 24%). This implies that over half the patients benefited from the noise-isolation intervention. In addition, significant improvements were seen in the key secondary endpoint of the incidence of moderate-to-severe pain within 48 h after surgery.

Preclinical animal studies have indicated that high-intensity noise can induce heightened pain sensitivity, substantially aggravating postincisional pain sensitization^13^. The present study and previous clinical investigations^14^ further suggest that noise triggers postoperative pain sensitization in surgical patients. Given that surgical trauma represents the phase of strongest nociceptive signalling, wearing noise-isolating headphones may mitigate noise-exacerbated pain sensitization during this critical window, thereby reducing postoperative pain risk. Building upon these mechanistic insights, the present study confirms that proactive intraoperative noise isolation is a safe, effective, and non-invasive preventive analgesic strategy, significantly enhancing postoperative pain management in patients after major abdominal surgery.

Among the secondary outcomes, maximum pain NRS scores and cumulative pain scores (both at rest and during movement) within 24 and 48 h after surgery were significantly lower in the noise isolation than control group. Maximum pain scores reflect the worst pain experience after surgery, whereas cumulative scores represent the overall pain burden. The reduction in both metrics indicates that intraoperative noise isolation provides comprehensive postoperative pain relief. Furthermore, the noise isolation group had a significantly lower probability of needing supplementary analgesics. Opioid-equivalent analgesic consumption also exhibited a trend towards a reduction in the noise isolation group. Reduced analgesic requirements imply not only less pain but also a lower risk of medication-related side effects. However, no statistically significant differences in adverse events were seen between the noise reduction and control groups, a finding potentially attributable to the multimodal analgesia protocol (combining nerve blocks with PCIA). Notably, the mean reduction in opioid-equivalent dose in the experimental group was modest (approximately 3.5 mg).

The multivariate analysis, which adjusted for baseline imbalances in surgical approach, confirmed that noise isolation was a strong, independent factor associated with reduced pain. The effect size for group assignment remained virtually unchanged and highly significant after adjustment. This stability strongly suggests that the observed benefit is a direct effect of the intervention, not a confounder of the different surgical practices. There was a minor but statistically significant imbalance in baseline ambient noise levels (MD 0.6 dB). Given that the intervention device provides 37 dB of noise reduction, an effect that is more than 60-fold greater than the baseline disparity, any residual confounding from this variable is considered negligible. Furthermore, our multivariate analysis confirmed the expected strong independent association between open surgery and a higher risk of severe pain, which aligns perfectly with the established surgical literature^18, 19^. This not only validates the statistical model but also accentuates the potency of the noise isolation intervention, which exhibited an effect that remained statistically significant even in the presence of such a robust clinical predictor. In summary, by using multivariate regression analysis to adjust for baseline confounders, this study has robustly demonstrated that noise isolation during general anaesthesia is an independent, statistically powerful factor associated with a significantly lower incidence of moderate-to-severe pain after surgery.

This multicentre study, encompassing diverse major surgeries (open/laparoscopic abdominal procedures), confirms a causal relationship between intraoperative noise exposure and adverse pain-related outcomes. It provides direct evidence that intervening on this modifiable factor improves clinical endpoints, further validating and deepening the theory that intraoperative noise significantly contributes to postoperative pain. Unlike pharmacological interventions, noise isolation does not involve pharmacological actions, avoiding the side effects of drugs themselves. Noise-cancelling headphones are low-cost, simple to operate, and easy to promote in hospitals at all levels, expanding the application scenarios and timing of non-pharmacological analgesia. This study focused on preventive intervention, targeting the initiation stage of central sensitization (during surgery) when pain originates, thereby reducing the postoperative pain burden at its source. This embodies the concept that prevention is superior to treatment. The results of this study have significant theoretical and practical importance, indicating that noise-cancelling headphones are a safe, simple, and non-invasive preventive analgesic strategy.

When interpreting the findings of the present study, the following limitations should be considered. First, despite randomization, slight baseline imbalances in surgical approach and ambient noise levels were observed. Although these were analytically addressed (as detailed above), they highlight a limitation in the randomization process. Second, all enrolled patients underwent abdominal surgery with PCIA for postoperative pain management. However, different surgical types may involve varying levels of noise and tissue trauma, and alternative analgesic approaches could influence pain outcomes. Thus, whether the observed effects of noise isolation on postoperative pain and analgesia translate to other surgical contexts or pain management regimens remains to be determined. Third, the absence of long-term outcomes, with observation limited to 48 h after surgery in this study, the impact of noise isolation on longer-term pain trajectories (for example, chronic postsurgical pain) remain unexplored.

For patients undergoing major abdominal surgery under general anaesthesia, intraoperative noise isolation using noise-cancelling headphones constitutes an effective, safe, straightforward, and non-invasive preventive intervention. Noise isolation significantly reduces the incidence of postoperative moderate-to-severe pain and should be incorporated as a valuable new component within perioperative multimodal analgesia strategies.

## Supplementary Material

zrag035_Supplementary_Data

## Data Availability

All data supporting this study are included in the article the *Supplementary material* contains methods only. Additional data are available from the corresponding author upon reasonable request.
