# Supplementary material for: Effect of noise isolation during general anaesthesia on the incidence of moderate-to-severe pain after major abdominal surgery: multicentre randomized clinical study
Source: BJS Open. 2026 May 28;10(3):zrag035. doi: 10.1093/bjsopen/zrag035 (PMC13217426; doi:10.1093/bjsopen/zrag035)
Supplement: zrag035_Supplementary_Data [file zrag035_supplementary_data.zip › Supplementary_Material.docx]

Study Number: 2023IIT110

**Effect of noise isolation during general anaesthesia on the incidence of moderate to severe pain after major abdominal surgery: multicentre randomized controlled study**

**Study Protocol**

| **Principle Invesitigator** |
| --- |
| Guangyou Duan, Ph.D. |
| Associate Researcher |
| Department of Anesthesia, Second Affiliated Hospital of Chongqing Medical University, No. 74 Linjiang Rd, Chongqing, China, duangy@hospital.cqmu.edu.cn |

**Table of Contents**

Synopsis and Study Schema --------------------------------------------------3

Abbreviations ------------------------------------------------------------------8

1.Study Objective --------------------------------------------------------------9

2.Study Design -----------------------------------------------------------------9

3.Study Participants -----------------------------------------------------------10

4.Randomization and Blinding/Unblinding ------------------------------11

5.Study Treatments -----------------------------------------------------------12

6.Administration of Study Drug and PCIA --------------------------------14

7.Concomitant Medications and Treatments ------------------------------14

8.Three stages of the study ---------------------------------------------------15

9.Study Evaluations and Endpoints -----------------------------------------22

10.Subject Completion/Withdrawal -----------------------------------------22

11.Adverse Event Reporting --------------------------------------------------23

12.Risk Assessment and Risk Management Plan --------------------------25

13.Ethical Principles -----------------------------------------------------------25

14.Data Quality Assuarance/Quality Control/ Management -------------26

15.Revision of the resrarch protocol-------------------------------------------27

16.Early termination of the study----------------------------------------------27

**Synopsis and Study Schema**

| Study Title | Effect of Noise Isolation During General Anesthesia on the Incidence of Moderate-to-Severe Postoperative Pain in Patients Undergoing Major Abdominal Surgery Study Protocol |
| --- | --- |
| Primary Objective | To demonstrate that noise isolation during general anesthesia reduces the incidence of moderate-to-severe postoperative pain (NRS ≥ 4) in patients undergoing major abdominal surgery under general anesthesia |
| Study Design | A Prospective, Single-Blind, Randomized, Controlled Trial |
| Research Center | Department of Anesthesia, Second Affiliated Hospital of Chongqing Medical University  Department of Anesthesia, Shapingba Hospital Affiliated to Chongqing University  Department of Anesthesia, Chongqing Iron and Steel Group General Hospital  Department of Anesthesia, The First Affiliated Hospital of Zhengzhou University |
| Number of Subjects | 304 |
| Study population | Inclusion Criteria:  i. Age between 18 and 70 years, regardless of sex.  ii. American Society of Anesthesiologists (ASA) physical status class I-III.  iii. Patients scheduled to undergo elective major abdominal surgery under general anesthesia (surgical duration ≥ 2 hours).  iv. Patients who voluntarily accept patient-controlled intravenous analgesia (PCIA) and provide written informed consent.  Exclusion Criteria:  i. Patients with significant pre-existing medical conditions or ASA physical status ≥ IV.  ii. Patients with auditory abnormalities.  iii. Patients requiring postoperative mechanical ventilation or receiving an epidural catheter or other types of regional anesthesia.  iv. Patients with preoperative chronic pain and/or long-term use of analgesic medications.  v. Patients unable to cooperate with the study for any reason.  Withdrawal Criteria:  i. Participants who voluntarily withdraw from the trial during the study.  ii.Participants with incomplete data collection that compromises efficacy evaluation. |
| Estimated duration of study | 1.5year (enrollment and follow-up) |
| Follow-up plan | **Preoperative Screening Period (Day -1 to Day 0):** ① Preoperative assessment ② Provide written informed consent ③ Review inclusion/exclusion criteria ④ Collect demographic characteristics (including sex, age, etc.) ⑤ Assess psychological status using the West China Mood Index (WCMI) ⑥ Instruct patients on using the Numerical Rating Scale (NRS) for pain assessment ⑦ Educate patients on operating patient-controlled intravenous analgesia (PCIA) pumps  **Anesthesia and Analgesia (Day 0):**  ① Monitoring: Routine monitoring including electrocardiography (ECG), pulse oximetry (SpO₂), heart rate (HR), and invasive arterial blood pressure (IBP). Bispectral Index (BIS) monitoring will be used continuously to assess depth of anesthesia until the end of surgery.  ② Anesthesia Administration: General anesthesia will be administered by experienced anesthesiologists. Both groups will receive identical anesthesia protocols.  Anesthesia Induction: Rapid sequence induction will be performed using:Midazolam: 0.04 mg/kg, Sufentanil: 0.3-0.5 μg/kg, Propofol: 2-2.5 mg/kg, Rocuronium: 0.6 mg/kg  Anesthesia Maintenance: Total intravenous anesthesia (TIVA) will be maintained with: Remifentanil infusion: 0.1-0.2 μg/kg/min, Propofol infusion: 4-12 mg/kg/h, Propofol will be titrated to maintain BIS values between 40-60.  Supplemental boluses of Sufentanil and Rocuronium will be administered as needed based on surgical requirements. Ondansetron 8 mg IV will be administered at the end of surgery.  ③ Study Intervention (Noise Isolation):  Experimental Group: Active noise-cancelling headphones will be placed on the patient after completion of anesthesia induction and maintained until the end of surgery to provide noise isolation.  Control Group: No headphones will be used during anesthesia induction, intubation, or surgery.  ④ Intraoperative Recording: Surgical duration and all administered medications will be recorded.  ⑤ Postoperative Regional Analgesia: At the end of surgery, patients will receive an ultrasound-guided bilateral transversus abdominis plane (TAP) block using 0.375% Ropivacaine Hydrochloride (20 mL per side).  ⑥ PCIA: Hydromorphone 0.15 mg/kg + 0.9% normal saline, total volume 150 mL. The PCIA pump was set with a 2 mL initial loading dose, a background infusion rate of 2 mL/h, a bolus dose of 2 mL, and a lockout time of 15 minutes.  ⑦ Additional analgesia was provided by the surgical team with non-steroidal anti-inflammatory drugs (NSAIDs), according to the clinical needs of the patients.  ⑧ Noise recording and analysis: This study utilized he AR844 noise meter by Sima Instrument (manufactured by Dongguan Wanchuang Electronic Products Co., Ltd., Dongguan, Guangdong, China) to monitor the noise levels. The noise meter recorded data at a frequency of once per second and had a measurable range of 0–130 decibels. It was used to continuously monitor the noise levels from the start of anesthesia (induction) to the end of anesthesia (cessation of anesthetic infusion). The noise meter measured the A-weighted sound levels (commonly referred to as ambient noise levels) during the entire period of general anesthesia. In this study, noise during general anesthesia was defined as the noise level from the start to the end of anesthesia. After data collection, the average A-weighted noise intensity during the entire general anesthesia period were calculated.  **Postoperative Hospitalization Follow-up Period (Day 0 to Postoperative Day 1):** ① Pain NRS score and administration of rescue analgesia prior to discharge from the Post-Anesthesia Care Unit (PACU). ② Pain NRS scores recorded during: 0-6 hours postoperatively, 6-12 hours postoperatively, 12-24 hours postoperatively, 24-48 hours postoperatively. ③ PCIA usage metrics during the same intervals (0-6h, 6-12h, 12-24h, 24-48h): Number of PCIA bolus attempts, PCIA analgesic consumption ④ Requirement for rescue analgesia during each specified postoperative interval. ⑤ Documentation of analgesia-related complications:Nausea and vomiting, Urinary retention, Dizziness, Intraoperative awareness (if reported). |
| Interventions | Experimental Group: Active noise-cancelling headphones will be placed on the patient after completion of anesthesia induction and maintained until the end of surgery to provide noise isolation.  Control Group: No headphones will be used during anesthesia induction, intubation, or surgery. |
| Outcomes | The primary outcome will be the incidence of moderate-to-severe pain (NRS≥ 4) within 24 hours postoperatively. Secondary outcomes will include:  The incidence of moderate-to-severe pain (NRS ≥ 4) within 48 hours postoperatively.  The area under the curve (AUC) of pain scores within 24 hours postoperatively.  Analgesic consumption at 24 hours and 48 hours postoperatively. This study aims to further clarify the impact of intraoperative noise isolation on postoperative pain.  Record postoperative pain and analgesic usage in both groups, including pain NRS scores and analgesic consumption. |

**Abbreviations**

| ASA | Anesthesiologists |
| --- | --- |
| PCIA | Patient Controlled Intravenous Analgesia |
| NRS | Numeric Rating Scale |
| ECG | Electrocardiography |
| SpO₂ | Pulse oximetry |
| HR | Heart rate |
| IBP | Invasive arterial blood pressure |
| BIS | Bispectral Index |
| TIVA | Total intravenous anesthesia |
| PACU | Post-Anesthesia Care Unit |
| AUC | Area under the curve |
|  |  |
|  |  |
|  |  |
|  |  |

1. **Study Objective**

To demonstrate that noise isolation during general anesthesia reduces the incidence of moderate-to-severe postoperative pain (NRS ≥ 4) in patients undergoing major abdominal surgery under general anesthesia.

1. **Study Design**

This study will be conducted as a **prospective randomized controlled clinical trial**. The protocol will be developed in accordance with the **CONSORT (Consolidated Standards of Reporting Trials) guidelines** for randomized controlled trials. Participants will be enrolled using a **sequential enrollment** approach.

**Randomization Method:**

**Simple randomization** will be employed. Random numbers will be generated using computer software. The randomization procedure will be implemented using the **sealed envelope method**. During the trial, an **independent researcher** will allocate participants to either the experimental or control group based on the random number inside a sealed envelope. **Odd numbers will be assigned to the experimental group; even numbers to the control group.** The envelopes will remain sealed until the completion of the entire study.

**Blinding:**

This study is designed as an **open-label trial**. **Blinding will be implemented where feasible:** All patients will be **blinded to their group assignment**. Data collection will be performed by **trained personnel** who are **not involved in the patient's anesthesia care** and are **blinded to group assignment**. Surgeons and anesthesiologists participating in the anesthesia and surgery **will not be involved** in the collection, entry, or analysis of study data.

1. **Study Participants**

**Inclusion Criteria:**

i. Age between 18 and 70 years, regardless of sex.

ii. American Society of Anesthesiologists (ASA) physical status class I-III.

iii. Patients scheduled to undergo elective major abdominal surgery under general anesthesia (surgical duration ≥ 2 hours).

iv. Patients who voluntarily accept patient-controlled intravenous analgesia (PCIA) and provide written informed consent.

**Exclusion Criteria:**

i. Patients with significant pre-existing medical conditions or ASA physical status ≥ IV.

ii. Patients with auditory abnormalities.

iii. Patients requiring postoperative mechanical ventilation or receiving an epidural catheter or other types of regional anesthesia.

iv. Patients with preoperative chronic pain and/or long-term use of analgesic medications.

v. Patients unable to cooperate with the study for any reason.

**Withdrawal Criteria:**

i. Participants who voluntarily withdraw from the trial during the study.

ii.Participants with incomplete data collection that compromises efficacy evaluation.

1. **Randomization/Blinding/** **Unblinding**
   1. **Randomization**

**Simple randomization** will be employed. Random numbers will be generated using computer software. The randomization procedure will be implemented using the **sealed envelope method**. During the trial, an **independent researcher** will allocate participants to either the experimental or control group based on the random number inside a sealed envelope. **Odd numbers will be assigned to the experimental group; even numbers to the control group.** The envelopes will remain sealed until the completion of the entire study.

- 1. **Blinding**

This study is designed as an **open-label trial**. **Blinding will be implemented where feasible:** All patients will be **blinded to their group assignment**. Data collection will be performed by **trained personnel** who are **not involved in the patient's anesthesia care** and are **blinded to group assignment**. Surgeons and anesthesiologists participating in the anesthesia and surgery **will not be involved** in the collection, entry, or analysis of study data.

**4.3 Unblinding**

Blindness can only be uncovered after statistical analysis is completed. Prior to this, any leakage of the blind bottom caused by non regulatory circumstances is called breaking of blindness. After breaking the blind, it is necessary to promptly record the time, reason, and personnel performing the early breaking, and notify the inspector as soon as possible. Once the blinding is broken in advance, the subject should generally not continue to participate in the study, and their experimental data cannot be used for efficacy evaluation analysis, but should still be included in the safety analysis dataset. At the same time, timely treatment and protection should be provided to the subjects.

1. **Study Treatments**

**5.1 The treatment of Anesthesia**

(1)Monitoring: Routine monitoring including electrocardiography (ECG), pulse oximetry (SpO₂), heart rate (HR), and invasive arterial blood pressure (IBP). Bispectral Index (BIS) monitoring will be used continuously to assess depth of anesthesia until the end of surgery.

(2)Anesthesia Administration: General anesthesia will be administered by experienced anesthesiologists. Both groups will receive identical anesthesia protocols. Anesthesia Induction: Rapid sequence induction will be performed using:Midazolam: 0.04 mg/kg, Sufentanil: 0.3-0.5 μg/kg, Propofol: 2-2.5 mg/kg, Rocuronium: 0.6 mg/kg; Anesthesia Maintenance: Total intravenous anesthesia (TIVA) will be maintained with: Remifentanil infusion: 0.1-0.2 μg/kg/min, Propofol infusion: 4-12 mg/kg/h, Propofol will be titrated to maintain BIS values between 40-60; Supplemental boluses of Sufentanil and Rocuronium will be administered as needed based on surgical requirements. Ondansetron 8 mg will be administered at the end of surgery.

**5.2 Study Intervention (Noise Isolation):**

(1)Experimental Group: Active noise-cancelling headphones will be placed on the patient after completion of anesthesia induction and maintained until the end of surgery to provide noise isolation.

(2)Control Group: No headphones will be used during anesthesia induction, intubation, or surgery.

**5.3 Intraoperative Recording**

Surgical duration and all administered medications will be recorded.

**5.4 Postoperative Analgesia**

**5.4.1 Postoperative Regional Analgesia**

At the end of surgery, patients will receive an ultrasound-guided bilateral transversus abdominis plane (TAP) block using 0.375% Ropivacaine Hydrochloride (20 mL per side).

**5.4.2 PCIA analgesia treatment**

(1) Preparation of PCIA pump

Hydromorphone 0.15 mg/kg + 0.9% normal saline, total volume 150 mL.

(2) Parameter settings of PCIA pump

initial loading dose: 2ml

Background infusion rate: 2 ml/h;

Bolus dose: 2ml;

Lock-in time:15 min;

**5.4.3 Additional analgesia**

Additional analgesia was provided by the surgical team with non-steroidal anti-inflammatory drugs (NSAIDs), according to the clinical needs of the patients.

1. **Administration of Study Drug**

This study is a physical intervention study. Apart from routine medications required for general anesthesia, no other drugs are involved.

**7. Concomitant Medications and Treatments**

During this trial, the use of any other medication is generally prohibited. Except for the drugs allowed during this trial, any other drugs used should be recorded in detail in the CRF, including the drug name, dosage, administration date, and indication for use.

1. **Three stages of the study**

Before conducting the study, the researcher will review and explain the written informed consent form (ICF) to each subject. Subjects who sign the ICF and undergo screening are eligible to participate in the study if they meet the inclusion criteria. The process of this study is divided into three stages: screening inclusion stage, baseline data collection stage and follow-up stage.

See TIME AND EVENTS SCHEDULE for detail.

- 1. **Screening inclusion stage**

Participants will be required to voluntarily sign an ICF before participating in this study, and participants who meet all inclusion criteria but do not meet any exclusion criteria will participate in the study.

**8.2 pre-study education and training stage**

During the initiation phase of the study, a dedicated researcher trained subjects on the NRS pain scoring guidelines and the use of PCIA pump.

1. NRS pain scoring guidelines

Numeric Rating Scale (NRS) were used to evaluate pain which were divided into 1-10 levels. According to the corresponding numbers, pain can be divided into different degrees, namely 0 level for no pain, 1-3 level for mild pain, 4-6 level for moderate pain, and 7-10 level for severe pain.

1. The use of PCIA pump.

A PCIA pump was provided by Jiangsu Renxian Medical Technology Co., Ltd.

①The importance of using PCIA pump to subjects: it can provide continuous pain relief; Compared to oral or intravenous drugs, PCIA pump can better control pain; It can reduce drug side effects; It can meet the individual needs of subjects and improve their satisfaction.

②The main structure of the electronic PCA pump is: injection pump; Automatic control device; Alarm device; Unidirectional valve (anti reflux); Infusion pipeline; Manual control; Tee joint.

③The principle of electronic PCIA pump: A specially designed drug storage pump is connected to the patient through a pipeline. Through this pump, analgesic drugs are continuously injected into the patient's body at a specific speed to achieve pain relief. The pump has a self-control button, which can be pressed by the patient to increase the injection amount when they feel pain.

④Safety: The continuous infusion speed, single addition volume and lockup time of the PCIA pump are regulated and managed by professional personnel; After turning on PCIA pump, analgesic drugs are pumped into the patient's body at a continuous and constant rate every hour. If the patient feels pain, they can press a single additional button, and no matter how many times the additional button is pressed within a fixed time, only one dose of medication is input; When there are abnormal situations such as blockages in the drug delivery pathway, the PCIA pump can alarm in time and restrict people other than anesthesiologists from modifying medication parameters, and prevent subjects from misoperating. If the patient has serious adverse reactions such as respiratory suppression and severe nausea and vomiting, follow-up personnel will adjust the parameters of the analgesic pump in time and stop using it if necessary according to clinical conditions.

- 1. **Follow-up stage**

(1) **Preoperative Screening Period (Day -1 to Day 0):**① Preoperative assessment
② Provide written informed consent
③ Review inclusion/exclusion criteria
④ Collect demographic characteristics (including sex, age, etc.)
⑤ Assess psychological status using the West China Mood Index (WCMI)
⑥ Instruct patients on using the Numerical Rating Scale (NRS) for pain assessment
⑦ Educate patients on operating patient-controlled intravenous analgesia (PCIA) pumps

(2) **Anesthesia and Analgesia (Day 0):**

① Monitoring: Routine monitoring including electrocardiography (ECG), pulse oximetry (SpO₂), heart rate (HR), and invasive arterial blood pressure (IBP). Bispectral Index (BIS) monitoring will be used continuously to assess depth of anesthesia until the end of surgery.

② Anesthesia Administration: General anesthesia will be administered by experienced anesthesiologists. Both groups will receive identical anesthesia protocols.

Anesthesia Induction: Rapid sequence induction will be performed using:Midazolam: 0.04 mg/kg, Sufentanil: 0.3-0.5 μg/kg, Propofol: 2-2.5 mg/kg, Rocuronium: 0.6 mg/kg

Anesthesia Maintenance: Total intravenous anesthesia (TIVA) will be maintained with: Remifentanil infusion: 0.1-0.2 μg/kg/min, Propofol infusion: 4-12 mg/kg/h, Propofol will be titrated to maintain BIS values between 40-60.

Supplemental boluses of Sufentanil and Rocuronium will be administered as needed based on surgical requirements. Ondansetron 8 mg IV will be administered at the end of surgery.

③ Study Intervention (Noise Isolation):

Experimental Group: Active noise-cancelling headphones will be placed on the patient after completion of anesthesia induction and maintained until the end of surgery to provide noise isolation.

Control Group: No headphones will be used during anesthesia induction, intubation, or surgery.

④ Intraoperative Recording: Surgical duration and all administered medications will be recorded.

⑤ Postoperative Regional Analgesia: At the end of surgery, patients will receive an ultrasound-guided bilateral transversus abdominis plane (TAP) block using 0.375% Ropivacaine Hydrochloride (20 mL per side).

⑥ PCIA: Hydromorphone 0.15 mg/kg + 0.9% normal saline, total volume 150 mL. The PCIA pump was set with a 2 mL initial loading dose, a background infusion rate of 2 mL/h, a bolus dose of 2 mL, and a lockout time of 15 minutes.

⑦ Additional analgesia was provided by the surgical team with non-steroidal anti-inflammatory drugs (NSAIDs), according to the clinical needs of the patients.

⑧ Noise recording and analysis: This study utilized he AR844 noise meter by Sima Instrument (manufactured by Dongguan Wanchuang Electronic Products Co., Ltd., Dongguan, Guangdong, China) to monitor the noise levels. The noise meter recorded data at a frequency of once per second and had a measurable range of 0–130 decibels. It was used to continuously monitor the noise levels from the start of anesthesia (induction) to the end of anesthesia (cessation of anesthetic infusion). The noise meter measured the A-weighted sound levels (commonly referred to as ambient noise levels) during the entire period of general anesthesia. In this study, noise during general anesthesia was defined as the noise level from the start to the end of anesthesia. After data collection, the average A-weighted noise intensity during the entire general anesthesia period were calculated.

(3) **Postoperative Hospitalization Follow-up Period (Day 0 to Postoperative Day 1):**
① Pain NRS score and administration of rescue analgesia prior to discharge from the Post-Anesthesia Care Unit (PACU).
② Pain NRS scores recorded during: 0-6 hours postoperatively, 6-12 hours postoperatively, 12-24 hours postoperatively, 24-48 hours postoperatively.
③ PCIA usage metrics during the same intervals (0-6h, 6-12h, 12-24h, 24-48h): Number of PCIA bolus attempts, PCIA analgesic consumption
④ Requirement for rescue analgesia during each specified postoperative interval.
⑤ Documentation of analgesia-related complications:Nausea and vomiting, Urinary retention, Dizziness, Intraoperative awareness (if reported).

**TIME AND EVENTS SCHEDULE**

| **Evaluation content** | **Screening inclusion stage** | **intraoperative** | **postoperative** | | | | |
| --- | --- | --- | --- | --- | --- | --- | --- |
|  |  |  | **PACU** | **6h** | **12h** | **24h** | **48h** |
| **Inclusion and Exclusion Criteria** | □ |  |  |  |  |  |  |
| **Informed Consent Form** | □ |  |  |  |  |  |  |
| **Subject Demographics** | □ |  |  |  |  |  |  |
| **West China Mood Index Scale** | □ |  |  |  |  |  |  |
| **Noise Monitoring** |  | □ |  |  |  |  |  |
| **BIS Monitoring** |  | □ |  |  |  |  |  |
| **Pain NRS Score** |  |  | □ | □ | □ | □ | □ |
| **Analgesia Pump Usage** |  |  |  | □ | □ | □ | □ |
| **Requirement for Rescue Analgesia** |  |  |  | □ | □ | □ | □ |
| **Nausea and Vomiting** |  |  |  | □ | □ | □ | □ |
| **Urinary Retention** |  |  |  | □ | □ | □ | □ |
| **Dizziness** |  |  |  | □ | □ | □ | □ |

1. **Study Evaluations and Endpoints**
   1. **Study Evaluations**
2. Efficacy

The incidence of moderate-to-severe pain (NRS ≥ 4) within 24 and 48 hours postoperatively; the accumulative postoperative pain NRS scores within 48 h after surgery, and the consumption of analgesic drugs within 24 and 48 h after surgery, The proportion of subjects requiring rescue analgesia within 48 hours after.

1. Safety

Percentages of subjects who had adverse events within 48 hours after surgery, such as nausea, vomiting, pruritus, constipation, dysuria, respiratory depression.

**9.2 Study Endpoints:**

Primary endpoint: the incidence of moderate-to-severe pain (NRS≥ 4) within 24 hours postoperatively.

Secondary and other endpoints: the incidence of moderate-to-severe pain (NRS ≥ 4) within 48 hours postoperatively; the accumulative postoperative pain NRS scores within 48 h after surgery, and the consumption of analgesic drugs within 24 and 48 h after surgery.

**10. Subject Completion/Withdrawal**

**10.1 Completion**

If the subject complete the evaluation within 48 hours of the follow-up phase, it is considered that treatment has been completed. If the subject terminate the study treatment for any reason before completing the evaluation within 48 hours of the follow-up phase, it will not be considered as having completed the treatment.

**10.2 Withdrawal from the Study**

Subject withdrawal from the trial refers to all subjects who have filled out the informed consent form and have been selected as qualified to enter the trial, and have encountered situations during the trial process where they are not suitable to continue the trial.

(1) Subjects experience severe adverse reactions (SAE) or were unable to tolerate surgical chemotherapy during the experiment.

(2) Subjects withdraw their informed consent form and requested to withdraw from this study.

(3) From a safety perspective, researchers need to discontinue the subjects' experiments.

(4) Subjects seriously violate the requirements of this experiment.

1. **Adverse Event Reporting**
   1. **Definition of adverse events (AE)**

The World Health Organization defines adverse events as injuries caused not by disease complications but by medical management related behaviors.

**11.2 Adverse event classification**

(1) Catastrophic events: An event of death or significant permanent loss of function (sensory, motor, physiological, or intellectual) unrelated to the subject's natural course or underlying condition.

(2) Severe events: An event of permanent physical impairment (sensory, motor, physiological, or intellectual) unrelated to the subject's natural course or underlying condition (unable to work or perform daily activities).

(3) Moderate events: The event of extending the hospitalization time or increasing nursing services for two or fewer subjects (Discomfort, reduced or impaired daily activities).

(4) Mild events: Events where there were no injuries, no extended hospital stay, or increased nursing services (Discomfort but does not interfere with daily activities).

**11.3 Record of adverse events in this study**

(1) Adverse events will be collected from signing the ICF and conducting research screening operations until the end of the study. During the experiment, an adverse event record form should be truthfully filled out, recording the occurrence time, severity, duration, measures taken, and resolution of the adverse event. If any clinical AE that meets the criteria for serious adverse events or catastrophic events occurs during the research process, it must be reported to Independent Ethics Committee (IEC) and medical management departments immediately. The researcher must fill out the SAE report form, send it to the SAE competent department, and file it with IEC of the research center.

(2) Adverse events should be recorded on the designated CRF adverse event table and fully described to determine the relationship between the adverse event and the investigational drug.

**12. Risk Assessment and Risk Management Plan**

The noise-canceling headphones used in this study serve as a physical intervention. Participants are patients undergoing major abdominal surgery, with head and neck surgeries explicitly excluded from the selection. Prior to the initiation of this trial, preliminary studies utilizing the same intervention method have been conducted, and no potential harms—such as local redness, swelling, or ischemia caused by ear cup pressure—were observed. Throughout the trial, the headphones will be correctly positioned and patient positioning closely monitored to prevent displacement and avoid compression of the auricle or scalp.

**13. Ethical Principles**

This clinical study must be conducted in accordance with the Helsinki Declaration and relevant Chinese clinical trial norms and regulations. Before starting the study, information including the research protocol and ICF must be submitted for approval by the Ethics Committee of the Second Affiliated Hospital of Chongqing Medical University.

Before the start of screening, researchers should have the responsibility to provide objective and comprehensive descriptions of purpose, treatments, and potential benefits and risks of the study to the subjects and their guardians, in order to make subjects aware of their right to decide whether to participate in the study and request withdrawal from the study. Researchers must obtain an ICF signed by each patient before entering the study, and explain the relevant regulations on personal information protection and privacy in the ICF, which should be kept as a research document. If changes need to be made to research documents such as ICFs during the research period, they must be reported to IEC for review and approval before implementation. If serious adverse events occur during the study period, they should be reported to IEC as required.

1. **Data Quality Assurance/Quality Control/** **management**

**14.1 Data Quality Assurance/Quality Control**

This study strictly follows the design plan and complies with relevant regulations to ensure the accuracy of research records and reports, and to ensure the reliability of data and the correctness of the research process. If there is any inconsistency with the research protocol during the experiment, it should be reported in a timely manner and appropriate measures should be taken.

- 1. **Data management**

The specific data management tasks include authorizing data management personnel to archive, process, and save the original recorded data (including paper and electronic versions of CRF) according to the research plan. The paper version of the CRF form is jointly filled out by the designated personnel and subjects, and should not be changed arbitrarily (in cases where changes are needed, the relevant instructions for CRF changes in this study should be followed). Research data should be strictly confidential in accordance with relevant national laws. The chief researcher is responsible for managing sealed data, and any situation involving data usage must be reviewed by the chief researcher and submitted to the research initiator for approval before implementation.

1. **Revision of the research protocol**

This research protocol was prepared by the main researchers based on a comprehensive review of the literature and approved by the institutional ethics committee before implementation. No one has the right to modify this protocol without formal amendment. If changes to the research protocol are required during the research period, they must be reported to IEC for review and approval before implementation. The use of the revised research protocol must be approved in writing by IEC.

1. **Early termination of the study**

If the subject is unable to continue participating in the study, please contact the researcher immediately and inform them of the current situation.

**Statistical Analysis Plan**

**Lead Statistician Qiang Chen, PHD Department of Statistics, College of Public Health, Chongqing Medical University, No. 61 Daxue City Middle Road, Chongqing, China, 401331**

**Table of Contents**

1. Abbreviations------------------------------------------------------------30

2. Introduction--------------------------------------------------------------31

3. Study overview---------------------------------------------------------33

3.1 Objectives

3.2 Trial design

3.3 Study participants

3.4 Samplesize

3.5 Randomization and blinding

3.6 Interventions

4. Study outcomes---------------------------------------------------------37

4.1 Primary and Secondary outcomes

5. Analysis data set--------------------------------------------------------38

6. Statistical analysis------------------------------------------------------39

6.1 General principles of statistical analysis

6.2 Baseline demographic and clinical characteristics

6.3 Outcome analysis

**1.Abbreviations**

| ASA | Anesthesiologists |
| --- | --- |
| PCIA | Patient Controlled Intravenous Analgesia |
| NRS | Numeric Rating Scale |
| ECG | Electrocardiography |
| SpO₂ | Pulse oximetry |
| HR | Heart rate |
| IBP | Invasive arterial blood pressure |
| BIS | Bispectral Index |
| TIVA | Total intravenous anesthesia |
| PACU | Post-Anesthesia Care Unit |
| AUC | Area under the curve |
|  |  |
|  |  |
|  |  |
|  |  |

**2. Introduction**

Noise is defined as unwanted, harmful sound that interferes with normal hearing, constituting an irregular and unpredictable combination of sounds. In the 1970s, intraoperative noise was first recognized and described as the third pollution in modern surgery. The World Health Organization (WHO) recommends that continuous background noise levels in hospitals should not exceed 35 dB, emphasizing the need to monitor sound levels in intensive care units and operating rooms. Over recent decades, with medical advancements and the introduction of sophisticated equipment, noise pollution in hospitals, particularly in operating rooms, has progressively increased. Numerous studies have found that noise levels during surgery frequently exceed WHO recommendations, with peaks reaching 120 decibels (dB). Operating room noise has been confirmed as a health hazard, adversely affecting both medical staff and surgical patients.

Previous research has established that general anesthesia does not interrupt the brain's perception of sounds and words. The central auditory pathways remain intact during general anesthesia, and patients' auditory cortices remain active. This indicates that although patients are unconscious under general anesthesia, they can still process auditory information. This theoretically explains patients' auditory perception of intraoperative noise during general anesthesia.

Building upon this foundation of auditory perception under general anesthesia, our preliminary clinical observations revealed a significant positive correlation between intraoperative noise levels and postoperative pain. Specifically, a greater proportion of time exposed to noise levels exceeding 70 dB during surgery was associated with higher Numerical Rating Scale (NRS) pain scores within 24 hours postoperatively (Chinese Clinical Trial Registry No.: ChiCTR2200057002).

It is reported that over 300 million surgical procedures are performed worldwide annually, with approximately 50% of patients experiencing moderate to severe postoperative pain. Postoperative pain increases the risk of complications, hinders early mobilization and recovery; thus, adequate postoperative analgesia remains a major goal and challenge in recovery management. With advances in anesthesiology, postoperative pain management has received increasing attention. Currently, opioid administration remains the primary analgesic method. However, the side effects of these drugs—such as urinary retention, pruritus, nausea, and vomiting—are often unavoidable. Consequently, many clinicians and researchers aim to adopt multimodal analgesia to reduce opioid dosages, for example, by combining non-steroidal anti-inflammatory drugs (NSAIDs) or nerve block techniques. Nevertheless, these approaches still carry inherent risks of side effects.

Therefore, in recent years, increasing research has focused on non-pharmacological, non-invasive patient interventions, such as acupuncture techniques and patient educational videos. Our previous randomized controlled trial (ClinicalTrials.gov ID: NCT05540691) further confirmed that the proportion of time with intraoperative noise ≥70 dB is an independent risk factor for higher postoperative pain scores. Providing patients with active noise-cancelling headphones during general anesthesia to isolate noise is a safe, inexpensive, and effective measure that can reduce postoperative pain and opioid consumption in patients undergoing abdominal laparoscopic surgery under general anesthesia.

Based on the above evidence, this study hypothesizes that noise isolation during general anesthesia can reduce the incidence of moderate-to-severe postoperative pain in patients undergoing major abdominal surgery. Consequently, this study plans to enroll patients undergoing major abdominal surgery under general anesthesia (surgical duration ≥ 2 hours) to determine, via a prospective, randomized, controlled, multicenter trial, whether intraoperative noise isolation can lower the incidence of moderate-to-severe postoperative pain and reduce analgesic requirements in this patient population.

**3. Study overview**

**3.1 Objectives**

To demonstrate that noise isolation during general anesthesia reduces the incidence of moderate-to-severe postoperative pain (NRS ≥ 4) in patients undergoing major abdominal surgery under general anesthesia.

To demonstrate that noise isolation during general anesthesia reduces postoperative opioid consumption in patients undergoing major abdominal surgery under general anesthesia.

**3.2 Trial design**

This was a prospective multicenter randomized controlled trial involving four tertiary hospitals in China: The Second Affiliated Hospital of Chongqing Medical University, The Shapingba Hospital, Chongqing University, The First Affiliated Hospital of Zhengzhou University, and Chonggang General Hospital. The protocol was formulated in accordance with the CONSORT guidelines for randomized controlled studies15, with participants enrolled sequentially at a single center. The study was Approved by the Ethics Committee (Approved NO.: (140) of 2023 [KELUN-SHEN]) and registered at ClinicalTrial.gov with the clinical trial registration number NCT06316440. The study will be conducted from April 2024 to May 2025. All patients provided informed consent before inclusion. The protocol for this research is attached as Appendix 1. The research data for removing patient information can be obtained through the email of the corresponding author upon reasonable requests.

**3.3 Study participants**

**(1) Inclusion criteria**

① age between 18 and 70 years, with no sex restrictions; ②ASA classification of grades I-III; ③ patients who underwent elective major abdominal surgery under general anesthesia (with an operation time of ≥2 hours); and ④ voluntary acceptance of PCIA and signing of the informed consent form.

**(2) Exclusion criteria**

① patients with a history of severe diseases and ASA grade ≥4; ② patients with abnormal hearing; ③ patients who required mechanical ventilation or received epidural catheters or other types of regional anesthesia after the operation; ④ patients with chronic pain before surgery and/or those who had been taking painkillers for a long time; ⑤ patients who were unable to cooperate with the research for any reason.

**(3) Elimination criteria**

① Patients who voluntarily terminated the trial during the research process; ② those whose incomplete data collection affected the judgment of validity.

**3.4 Sample size**

This study uses the incidence of moderate-to-severe pain within 24 hours postoperatively as the primary outcome. Based on preliminary clinical observations, the incidence of moderate-to-severe pain within 24 hours after abdominal surgery is approximately 50%. We hypothesize that this incidence will decrease to 30% with noise isolation intervention.

Using PASS 2021 software (Power Analysis and Sample Size, NCSS LLC) a two-arm parallel superiority trial with a 1:1 allocation ratio, a significance level (α) of 0.05, and statistical power (1-β) of 0.90. The minimum required sample size is 121 patients per group. Accounting for an estimated 20% attrition/dropout rate, the study requires enrollment of at least 152 patients per group. Therefore, the total planned sample size is 304 patients.

**3.5 Randomization and Blinding**

The protocol will be developed in accordance with the CONSORT (Consolidated Standards of Reporting Trials) guidelines for randomized controlled trials. Participants will be enrolled using a sequential enrollment approach. Simple randomization will be employed.Random numbers will be generated using computer software.The randomization procedure will be implemented using the sealed envelope method. During the trial, an independent researcher will allocate participants to either the experimental or control group based on the random number inside a sealed envelope.Odd numbers will be assigned to the experimental group; even numbers to the control group.The envelopes will remain sealed until the completion of the entire study.

This study is designed as an open-label trial. Blinding will be implemented where feasible: All patients will be blinded to their group assignment. Data collection will be performed by trained personnel who are not involved in the patient's anesthesia care and are blinded to group assignment. Surgeons and anesthesiologists participating in the anesthesia and surgery will not be involved in the collection, entry, or analysis of study data.

**3.6 Interventions**

Experimental Group: Active noise-cancelling headphones will be placed on the patient after completion of anesthesia induction and maintained until the end of surgery to provide noise isolation.

Control Group: No headphones will be used during anesthesia induction, intubation, or surgery.

**4. Study outcomes**

**4.1 Primary and Secondary endpoint**

The primary outcome indicator of this study was the incidence of moderate to severe pain (NRS ≥ 4) within 24 h after surgery. The secondary outcome indicators included the incidence of moderate to severe pain within 48h after surgery, the accumulative postoperative pain NRS scores within 48 h after surgery, and the consumption of analgesic drugs within 24 and 48 h after surgery.

The observation indicators were the maximum pain NRS score experienced by the patient within 48 h after surgery (ranging from 0 to 10, where 0 indicates no pain at all and 10 indicates the most severe pain). The higher the score, the more severe the pain). The researchers followed up with the patients at 6, 12, 24, and 48 h after surgery to record the maximum static and dynamic pain experienced by the patients in the 0-6 hour, 6-12 hour, 12-24 hour, and 24-48 hour periods. Static pain refers to pain experienced while lying still in bed, whereas dynamic pain refers to pain experienced during activities such as turning over or coughing. The researchers recorded the maximum pain NRS score for each period of time. Other observation indicators mainly included general demographic parameters before surgery, the patient's emotional state, ASA classification，the name of the surgery, the duration of the surgery, intraoperative medication use, postoperative nausea, vomiting, urinary retention, and dizziness.

**5. Analysis data set**

The principle of Intention To Treat (ITT) analysis is that the analysis should include all randomized subjects, that is, all subjects originally planned for treatment need to be included in the analysis, rather than based on actual completed subjects. The analysis conducted according to this principle is the best analysis, and the result is that every subject randomly assigned to the intervention or control group should be fully followed up and the research results (such as efficacy and safety evaluations) should be recorded, regardless of their compliance.

According to the ITT principle, there are three types of data that can be adopted for statistical analysis:

FAS (Full Analysis Set) refers to the ideal population of participants who are as close as possible to the ITT principles. It should include almost all randomized subjects. Only those who were excluded during the import period but were not enrolled or did not have any follow-up data after enrollment can be excluded from the FAS population. (i.e.subjects who have taken the medication once and tested for efficacy once should be included in FAS).

PPS (Per Protocol Set) is a subset of the full analysis set, in which subjects have good compliance and do not violate the protocol. The baseline values of the main indicators are complete.

SS (Safety Analysis Set) safety dataset: It should include all randomized subjects who received at least one treatment and had at least one comprehensive evaluation.

In this study, the analysis of the main and secondary outcomes was based on the FAS and PPS datasets, and conclusions were drawn mainly from the FAS dataset. The number of participants who were enrolled, completed the experiment, and withdrew from the experiment will be summarized. List the participants who were not included and the reasons for their exclusion.

**6.Statistical analysis**

- 1. **General principles of statistical analysis**

This study is a statistical analysis plan developed with the participation of a professional statistician. All statistical analyses were conducted using the statistical software package (IBM SPSS Statistics 25). All tests of statistical significance are two-sided, and a P value of <0.05 will be considered statistically significant (unless otherwise specified).

Continuous variables will be represented as mean, standard deviation (normal distribution), or median (interquartile spacing). The classification variables will be expressed in numbers and percentages. Baseline demographic and clinical characteristics will be displayed and compared between the intervention group and the control group. The differences in continuous variables will be compared using the Student t-test for normally distributed variables or the Mann Whitney U-test for non normally distributed variables. Comparisons on qualitative variables will be done using the χ2 test or Fisher’s exact test.

**6.2 Baseline demographic and clinical characteristics**

Baseline characteristics will be compared between intervention and control group to evaluate the comparability, including Age, Gender, Height, Weight, BMI, ASA, HEI, Surgery duration, Blood loss, Intraoperative remifentanil and sufentanil, Mean noise intensity, Surgery type, Surgery method, Cancer surgery, Study sites.

Use Fisher's exact test for data represented by the percentage of participants (%). Use Mann Whitney U-test for data represented by median (IQR). Use Student's t test for data represented by mean (SD).

**6.3 Outcome analysis**

The primary outcomes were compared according to the modified intention to-treat principle and included those patients who had provided consent, undergone randomization, and completed the 48-hour postoperative follow-up. The incidence of moderate-to-severe pain within 24 h after surgery was compared with a Chi-χ2 test, with differences between groups expressed as relative risk and 95% confidence interval (CI). A post hoc sensitivity analysis was also performed through imputing the missing primary endpoint data. The worst outcome to participants in the noise isolation group was assigned.

The secondary and other outcomes were analyzed based on the data attribute. Continuous variables were compared using an independent *t* test or Mann-Whitney test, and categorical variables were analyzed using Chi-χ2 test, continuity corrected χ2 test or Fisher’s exact test. Mean differences (MD) or relative risks (RR) with 95% CI were also calculated. In addition, univariate logistic regression analysis was performed to determine effects of sex (female *vs.* male), age group (≥60 *vs.* <60), BMI group (≥28 *vs.* <28), ASA grade (III *vs.* II), Surgery type (Gynecology *vs*. Gastrointestinal), HEI (≥9 *vs.* <9), Surgery method (open *vs.* endoscope), Cancer surgery (yes *vs.* no) and Group (noise isolation *vs.* control) on incidence of moderate-to-severe pain within 24 h after surgery. And multiply logistic regression analysis was then performed using the factors with P values less than 0.1. Subgroup analysis was also performed to compare the differences between noise isolation and control groups.
