# Supplementary material for: Effect of noise isolation during general anaesthesia on the incidence of moderate-to-severe pain after major abdominal surgery: multicentre randomized clinical study
Source: BJS Open. 2026 May 28;10(3):zrag035. doi: 10.1093/bjsopen/zrag035 (PMC13217426; doi:10.1093/bjsopen/zrag035)
Supplement: zrag035_Supplementary_Data [file zrag035_supplementary_data.zip › Cover letter0125.docx]

Jan 25, 2026

To the Editorial Board,
**BJS Open**

Dear Editors,

We are pleased to submit our original research manuscript, **"Effect of noise isolation during general anesthesia on the incidence of moderate to severe pain after major abdominal surgery: A multicenter randomized controlled study"** for your consideration for publication in **BJS Open**.

Despite advances in perioperative care, postoperative pain remains a major clinical challenge, with 40%–60% of patients globally still experiencing moderate-to-severe pain. Our study introduces and evaluates a novel, non-pharmacological intervention—**intraoperative noise isolation using noise-canceling headphones**—as a preventive analgesic strategy. The findings from our multi-center, randomized clinical trial demonstrate that this simple, safe, and low-cost intervention **significantly reduces the incidence of moderate-to-severe pain by more than 25%** within the first 24 and 48 hours after major abdominal surgery. Furthermore, it led to a substantial reduction in analgesic consumption and improved patient-reported pain scores at rest and during movement.

We believe our manuscript is highly appropriate for **BJS Open** for the following reasons:

1) **High Public Health Relevance:** Ineffective pain management is a pervasive problem that prolongs recovery, increases healthcare costs, and diminishes patient satisfaction. Our study addresses this issue with a scalable solution.
2) **Innovation and Practical Impact:** To the best of our knowledge, this is the first large-scale, multi-center trial to rigorously assess the role of intraoperative noise isolation as a direct component of multimodal analgesia. Our findings argue for its integration into standard ERAS (Enhanced Recovery After Surgery) protocols.
3) **Alignment with Journal Scope:** The journal's focus on publishing high-impact clinical research that influences practice across multiple medical disciplines aligns perfectly with our work, which has immediate implications for anesthesiologists, surgeons, and perioperative physicians worldwide.

I would like to declare on behalf of my co-authors that the work described was original research that has not been published previously, and is not under consideration for publication elsewhere, in whole or in part. All the authors listed have approved the manuscript that is enclosed. No conflict of interest exists in the submission of this manuscript, and the manuscript is approved by all authors for publication. The trial was registered at ClinicalTrial.gov with the clinical trial registration number NCT06316440 and was approved by the institutional review boards of all participating centers. Written informed consent was obtained from all participants.

We deeply appreciate your consideration of our manuscript, and we look forward to receiving comments from the reviewers. If you have any queries, please don’t hesitate to contact me at the address below. Thank you and best regards!

Yours sincerely,

Guangyou Duan

Corresponding author: The Second Affiliated Hospital, Chongqing Medical University, No. 74, Linjiang Road, Yuzhong District, Chongqing, China. Tel +86-18323376014, Email: duangy@hospital.cqmu.edu.cn.
